# Design and Fabrication of a Push-Pull Electrostatic Actuated Cantilever Waveguide Scanner

**DOI:** 10.3390/mi10070432

**Published:** 2019-06-29

**Authors:** Wei-Chih Wang, Kebin Gu, ChiLeung Tsui

**Affiliations:** 1 Department of Mechanical Engineering, University of Washington, Seattle, WA 98185, USA; 2 Department of Electrical and Computer Engineering, University of Washington, Seattle, WA 98185, USA; 3 Institute of Nanoengineering and Microsystems, National Tsinghua University, Hsinchu 300, Taiwan; 4 Department of Power Mechanical Engineering, National Tsinghua University, Hsinchu 300, Taiwan

**Keywords:** cantilever waveguide, electrostatic actuator, non-resonating scanner, optical scanner, push-pull actuator, rib waveguide

## Abstract

The paper presents a novel fully integrated MEMS-based non-resonating operated 2D mechanical scanning system using a 1D push-pull actuator. Details of the design, fabrication and tests performed are presented. The current design utilizes an integrated electrostatic push-pull actuator and a SU-8 rib waveguide with a large core cross section (4 μm in height and 20 μm in width) in broadband single mode operation (λ = 0.4 μm to 0.65 μm). We have successfully demonstrated a 2D scanning motion using non- resonating operation with 201 Hz in vertical direction and 20 Hz in horizontal direction. This non-resonating scanner system has achieved a field of view (FOV) of 0.019 to 0.072 radians in vertical and horizontal directions, with the advantage of overcoming its frequency shift caused by fabrication uncertainties. In addition, we observed two fundamental resonances at 201 and 536 Hz in the vertical and horizontal directions with corresponding displacements of 130 and 19 μm, or 0.072 and 0.0105 radian field of view operating at a +150 V input. A gradient index (GRIN) lens is placed at the end of the waveguide to focus the diverging beam output from the waveguide and a 20 μm beam diameter is observed at the focal plane. The transmission efficiency of the waveguide is slightly low (~10%) and slight tensile residual stress can be observed at the cantilever portion of the waveguide due to inherent imperfections in the fabrication process.

## 1. Introduction

Modern day micro-scale imaging and display systems utilizes miniature scanner technologies for capturing images and displaying high density information in a small working environment. Advanced imaging systems, such as scanning confocal microscopy use micro scale scanners for real-time sub-cellular resolution imaging [1,2,3,4]. Portable video projection systems use miniature scanners for displaying high resolution contents with a light-weight form factor and low power requirement. [5,6,7,8,9]. Virtual displays found in head-mounted displays (HMD) systems uses scanners to display large amount of information in a small display area for augmented reality/virtual reality applications [10,11,12,13,14]. Most of the miniature scanner system utilizes MEMS scanning mirrors, which requires the mirrors and the deflecting components to be significantly larger than the input beam diameter to avoid clipping or creating additional diffractions at the output. Furthermore, the size of the conventional display system is proportional to the resolution and/or the field of view (FOV) of the device, which severely limits the possibility of reducing system footprint without making a compromise on the device specification.

To overcome the minimum size restrictions of mirror-based scanner systems without sacrificing resolution and FOV, an optical scanner based on an electromechanically deflected micro-fabricated waveguide has been previously developed by our research team [15,16,17,18,19,20]. A pair of commercially available lead zirconate titanate (Pb[Zr(x)Ti(1-x)]O3) (PZT) actuators drives the 2D motion of the waveguide. The design is able to steer the coupled optical beam via the deflection of the waveguide; however, the design still has a relatively large footprint (each actuator is 20 mm × 4.8 mm × 0.6 mm and they are arranged perpendicularly) compared to its output beam deflection angle. Also, the assembly process is challenging, time consuming, and inconsistent due to the combined use of off-the-shelf parts and micro-fabricated waveguide. The output of the scanner is also inconsistent after each system reset due to the assembly process.

**Figure 1:**
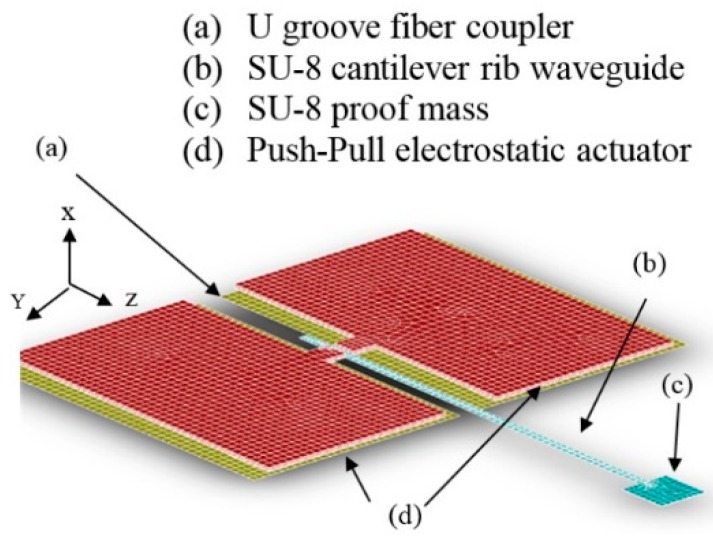
Schematic of the electrostatic MEMS scanner.

In this paper, design improvements to the micro-fabricated waveguide system is presented (Figure 1). To increase the robustness of the scanner system in the new design, a MEMS-based push-pull actuator is fully integrated with the micro-fabricated waveguide. The integrated actuator reduces the rigid length of the device and increases the flexibility of the overall system, allowing it to work in tight spaces without invading the surroundings. The new design also allows the incorporation of the light source and the scanner probe in a single package, thus reducing the overall system size. Compared to off-the-shelf actuators, the MEMS-based push-pull actuator provides a better signal to noise ratio and consumes relatively lower power during operations. Finally, the push-pull actuator mechanism will be able to provide a 2D scanning motion via 1D actuation, which reduces the complexity of the scanner and the footprint of the entire system.

Most of the resonant scanner designs reported so far, including our previous designs, operate at resonance to achieve high line resolution and high FOV [15,16,17,18,19,20,21,22]. However, this design presents several challenges, such as achieving a large frequency difference between the two resonant operating frequencies. A larger deflection angle of the high-frequency mode is usually harder to achieve due to damping in the system. In addition, reducing the frequency of the low-frequency mode would require altering materials or geometry of the scanner. Thus, frequency reduction can only be achieved by sacrificing the device compactness [15]. Furthermore, the final frequency of operation is highly dependent on the fabrication process. 

In this paper, we report a new driving scheme that can potentially overcome the above issues by operating one of the two directions at an extremely low non-resonant frequency (>20 fps) while maintaining a high resonant frequency in the other direction. The non-resonant frequency operation relaxes the restriction of the lower operating frequency and further improves the operating condition. The high resolution of the device can then be obtained through the large difference of the two operation frequencies. The deflection angle or FOV can also be optimized. In the following section, we will first examine the design and operational principles of this novel scanner through finite element analysis (FEA) using ANSYS (ANSYS, Canonsburg, PA., USA), CoventorWare and Rsoft (RSoft Design Group, Inc., Ossining, NY, USA) followed by a description of the fabrication process and results from the optical performance.

## 2. Design and Operation Principles

### 2.1. Operation Principles

The design of the scanner uses the 1D actuation of a pair of “push–pull” actuators to generate 2D scanning motion on a micro-fabricated cantilever waveguide (Figure 1). For image acquisition, the same scanning waveguide is used to capture and channel the backscattered to a photodetector. For display operation, this step is not needed. A long, slender SU-8 structure is placed between the two actuators to serve as the waveguide for the scanner. A coupler is placed at the input end of the SU-8 waveguide to couple the light from an optical fiber to the cantilever waveguide. The optical fiber is placed in a U-shape fiber groove to properly align it with the coupler. A proof mass is attached at the output end of the SU-8 waveguide for reducing the tip displacement of the microfabricated SU-8 waveguide without affecting the output beam deflection angle. The waveguide structure is connected to the actuators by the rotating arm. The two ends (in the z-direction) of rectangular-shaped actuating pads in Figure 1 are anchored to the substrate leaving the middle of the actuator suspended and connected to the rotating arm. This push-pull actuator configuration can be used with piezoelectric, electrostatic, electromagnetic, or magnetostrictive actuation methods. In this paper, a parallel-plate MEMS electrostatic actuator is presented.

**Figure 2:**
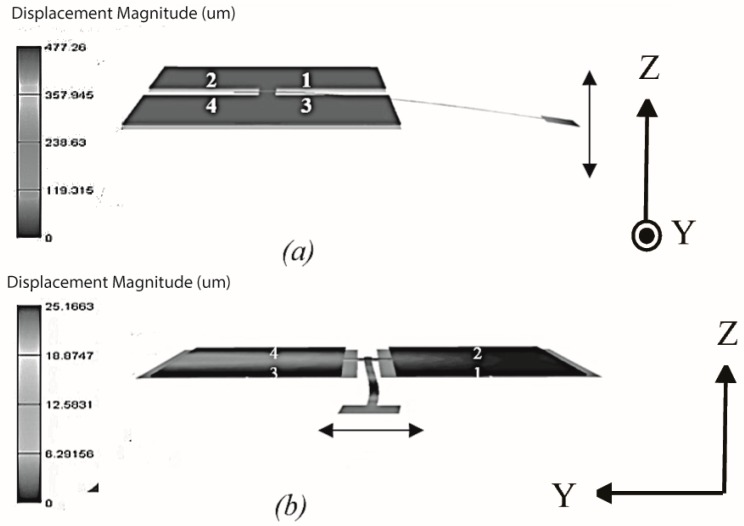
ANSYS harmonic virtualization of a mechanical resonating scanner. (**a**) Low-frequency motion under the excitation of the first mode along the z-axis, and (**b**) high-frequency motion under the excitation of the second mode along the y-axis.

The basic operating principle of this 2D scanner is shown in Figure 2 [22]. The scanner motion is generated by the pair of push-pull electrostatic actuators. The out-of-plane buckling of the four actuating pads generates bending and twisting motion on the rotating arm. The resulting bending and twisting on the rotating arm causes the attached waveguide to move in or out of plane. For out-of-plane waveguide motion (Figure 2a), actuator pads 1 and 2 (or actuator pads 3 and 4) must be actuated in the same direction, magnitude and phase. This will create vertical motion at the rotating arm due to the actuators hinging on the fixed ends. For horizontal waveguide motion (Figure 2b), actuator pads 1 and 3 (or actuator pad 2 and 4) must be driven in the same direction, magnitude and phase. This will produce rock the rotating arm side to side, causing the waveguide to move horizontally. The simultaneous excitation of the waveguide in the horizontal and vertical direction with the superposition of waveforms at the correctly designed frequencies and/or phase will drive the waveguide in Lissajou or raster scanning motion. 

Lissajou and raster scanning pattern is used instead of spiral scanning pattern due to the geometry of the micro-fabricated waveguide. The high aspect ratio of the rectangular cross-sectioned beam (cross-section width is ~5 times its thickness) makes it difficult to achieve spiral scanning motion. For spiral scanning pattern, a circular cladded waveguide is the preferred geometry, which is challenging to fabricated using the planar process in typical micro-fabrication. Additionally, a comparative large cladding (>50 µm) is required to confine the wave and maintains a single mode operation at the require geometry (~few µm for the core) in a waveguide with uniform cladding. Thus, to eliminate the need of a large cladding to create a waveguide core that has similar size to an optical fiber core, the rib waveguide geometry is employed in our design. The rib waveguide is a two-layer waveguide structure that consists of a rib layer on top of a wider slab layer, and the careful optimization of the geometric ratio between the two layer is necessary to achieve the desirable mechanical performance and optical output. 

### 2.2. Mechanical Design and Simulation 

The scanning FOV and line resolution are two critical parameters that needs to be studied to optimize the scanner system performance. A large FOV can be obtained when the displacement and the angle of rotation for the waveguide is maximized at its resonant frequencies and higher line resolution can be achieved by increasing the ratio of the horizontal and vertical operating frequencies. Thus, the design of the scanner needs to allow the system to operate at two distinctly different resonant frequencies in the horizontal and vertical directions. 

The components of the system were analyzed individually to obtain optimized dimensions for achieving the best line resolution and FOV. The critical parameters that needed to be analyzed were (1) length, width, and height of the proof mass, (2) the width, thickness, and length of the waveguide, and (3) the geometry, layout, and input voltage of the actuator. Both analytical and numerical analyses were performed and compared.

**Figure 3:**
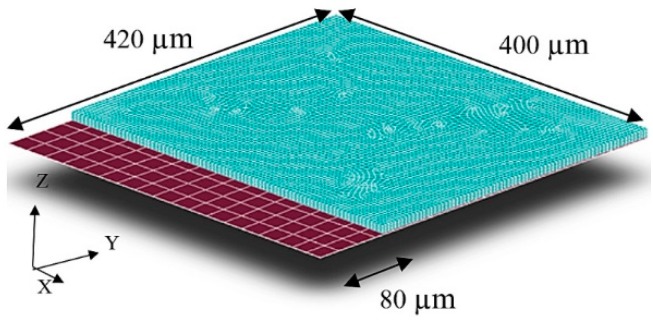
The modified parallel plate actuator. The bottom electrode is expanded along the y-axis so that the fringe effect causes larger electrostatic force in the y direction than in the x direction.

The modified parallel plate actuator was used in the scanner design. The conventional capacitive actuator was either a set of parallel plates or a set of comb drives [23]. However, the driving force was dominant in one direction and negligible in the other two directions. Figure 3 shows the modified actuator design with the extended bottom electrode. The non-equivalent electrodes increase the utilization of electrostatic fringe effect. Therefore, a larger in-plane electrostatic force was generated in the y direction. The FEM results show that an input of 20 V to the modified actuator results in a 27.66 µN out-of-plane reaction force (Fz). In the two in-plane directions, Fy was 8.64 × 10^−2^ µN, which was over two orders of magnitude larger than Fx of 1.92 × 10^−4^ µN. The model used slightly different dimensions to generate the subsequent FEM harmonic response of the scanner. 

**Figure 4:**
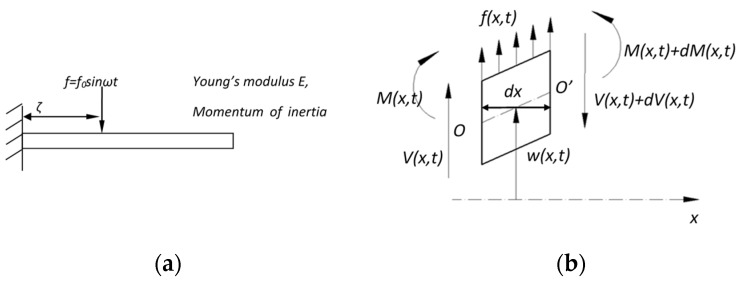
(**a**) A beam is subject to the external force f and (**b**) free-body diagram of an element of a beam.

For the cantilever waveguide mechanical model, both analytical and numerical analyses were performed. The analytical model uses the Timoshenko beam model to estimate the deflection angle of the cantilever waveguide (Figure 4a). Figure 4b shows the free-body diagram of an element of a beam, where M(x,t) is the bending moment; V(x,t) is the shear force; w(x,t) is the displacement of the vibration; and f(x,t) is the external force per unit length.

The displacement of the beam can be determined using the mode superposition principle and is written as [24]:

(1) $$w(x,t)=\sum_{n=1}^{\infty }W_{n}(x)q_{n}(t),$$

where *W_n_(x)* is the n-th normalized mode shape and *q_n_(t)* is the generalized coordinate in the n-th mode. For the fixed-free boundary condition as shown in Figure 4a, *W_n_(x)* and *q_n_(t)* can be calculated in (2) and (3), respectively [24]:

(2) $$W_{n}(x)=C_{n}\left[\sin\beta_{n}x-\sinh\beta_{n}x-\alpha_{n}\left(\cos\beta_{n}x-\cosh\beta_{n}x\right)\right]$$

(3) $$\begin{array}{ll} \frac{d^{2}q_{n}(t)}{dt^{2}}+\omega_{n}{}^{2}q_{n}(t) & =\int f_{0}\delta (x-\zeta )\sin\omega tW_{n}(x)dx\\ & =f_{0}\sin\omega tW_{n}(\zeta ) \end{array},$$

where

$$\beta_{n}{}^{4}=\frac{\omega_{n}{}^{2}}{c^{2}}=\frac{\rho A\omega_{n}{}^{2}}{EI}$$

$$\alpha_{n}=\frac{\sin\beta_{n}l+\sinh\beta_{n}l}{\cos\beta_{n}l+\cosh\beta_{n}l}.$$

*W_n_(x)* is determined by boundary conditions, material properties and resonant frequencies. *q_n_(t)* is subject to the change of the external force *f* and *W_n_(ζ)*. To achieve large deflection angle which is proportional to *dw(x,t)/dx*, the most straightforward strategy is to increase *f_0_* and *W_n_(ζ)*:

1. *f_o_* can be increased by increasing the driving electrostatic force.
2. To increase *W_n_(ζ)*, if the device is operating at the first resonance frequency, *W(x)* increases as *x* increases. *ζ* should be far away the fixed end to increase *W_n_(ζ)*. However, due to the limitation of the overall length of the MEMS device, *ζ* cannot be too far from the fixed end. If the device is operating at the higher resonant frequency, the force should not be applied to the nodal point, where *W_n_(ζ)* is equal to zero.

**Figure 5:**
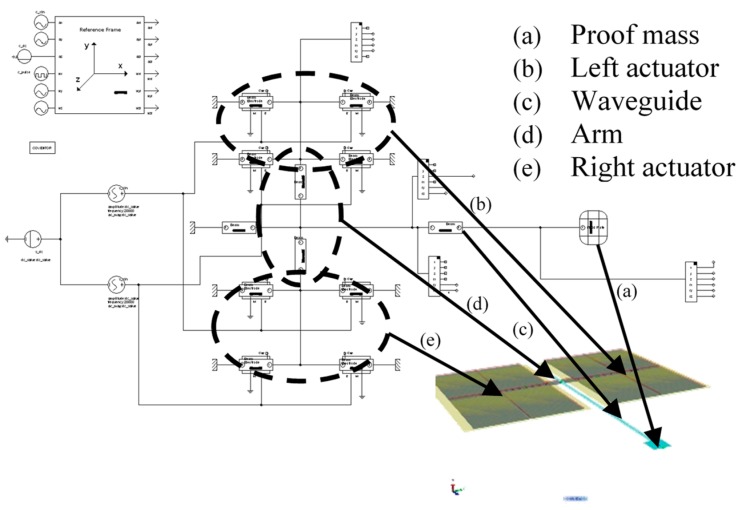
The 3D physical geometry of the scanner mapped to the corresponding behavior symbols.

The overall scanner design was also analyzed numerically using Architect3D in CoventorWare for optimization. The system-level model method can significantly reduce the simulation time compared to traditional finite element methods (FEM) while maintaining relatively high accuracy. The schematic layout modeled in Architect3D and the corresponding 3D model is shown in Figure 5, it included components such as beams, beams with electrodes, rigid plate, anchors, bus connectors, reference frames and signal sources. These components modeled the mechanical and electrical behaviors of the scanner during the optimization process.

The optimization of the scanner involved tuning the devices parameters so that the FOV and the line resolution of the scanners are optimized. The line resolution of the device is maximized by increasing the difference between the two operation frequencies. 

The resonant frequency of the high-frequency mode was designed so that it was 200 times larger than the resonant frequency of the low-frequency mode in order to obtain the highest scanning resolution. In our current experimental setup, it was difficult to drive a device above 20 kHz. Therefore, resonant frequency of the low-frequency mode was limited to less than 100 Hz. The field of view was maximized for the scanning direction that operates at the lower resonant frequency.

The resonant frequency of a cantilever beam is a function of its mass and spring constant, thus the operating frequency can be reduced by increasing the length of the waveguide or by incorporating a large proof mass. However, a waveguide design with excessive length is going to compromise the compactness of the device. Therefore, the final design for the length and width of the waveguide is 2250 µm and 55 µm, respectively, and the size of proof mass is 300 μm × 300 μm × 24 μm. The thickness of the slab and the ridge are both 2 µm. The modal analysis results obtained from Architect are verified by FEM and analytical results (Table 1) and the rotational angle for each of the obtained modes is optimized.

**Figure 6:**
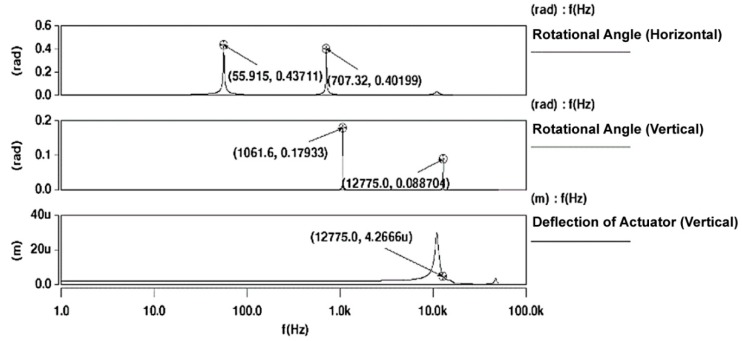
Architect3D in CoventorWare simulation results: (**top** and **middle**): deflection angle as a function of frequency and (**bottom**): deflection response of the actuator.

The harmonic response of the scanner design is studied by applying electrostatic actuation to excite vertical (at lower operating frequency) and horizontal motion (at higher operating frequency) in the waveguide as shown in Figure 2. Air damping factor is also applied to the simulation based on the measurement from a fiber viscometer [25]. The rotational angle response for the waveguide motion in both directions are shown in Figure 6. For vertical motion (the top trace), two rotational angle peaks are observed. To maximize the FOV, the lower frequency (55.9 Hz) is chosen as the designed operating frequency with a rotational angle of 0.44 rad. In the vertical direction, two peaks are also observed in the response (the middle trace) and the 12,775 Hz peak is chosen as the high-frequency mode (rotational angle is 0.089 rad) to maximize the line resolution. The bottom trace shows the deflection response of the electrodes and it can be observed that the electrodes achieves a maximum of 4.27 µm at the high frequency operation mode. This means that the actuators are able to operate without interference with the designed gap space (20 µm) between the parallel plates.

Although the simulation shows promising line resolution and FOV, there were challenges found in fabrication and in tests performed of the resonating design. The main obstacle is apparently on overcoming the weight of the proof mass at the tip of the cantilever. It was found later that SU-8 cantilever is much softer than the model based on the published material properties. Several design changes were made including the proof mass thickness and the no-resonating design to further simplify the fabrication and operation. 

**Figure 7:**
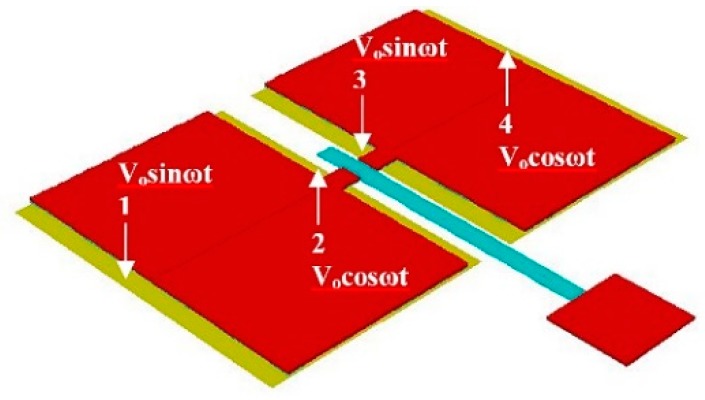
The optimized scanner design with a proof mass of 60 µm × 60 µm × 4 µm, waveguide length of 1820 µm and a width of the slab of 20 µm.

For the non-resonating scanner design, the same parametric study to optimize the design was also performed using Architect3D in CoventorWare. The modified actuator shown in Figure 7 is used to increase the in-plane electrostatic force, leading to a larger FOV. When the device is in operation, Electrode 1 and 3 are applied with the same waveform function of *V_o_sinωt* and Electrode 2 and 4 are applied with a *V_o_cosωt*, where *ω* is the resonant frequency of the high-frequency mode and *V_o_* is the amplitude of the input voltage. For low frequency direction, all electrodes were driven by a same wave function with lower frequency. Suppose the waveguide is simultaneously driven at the above two frequencies, a raster scanning motion will appear at the distal end. 

**Figure 8:**
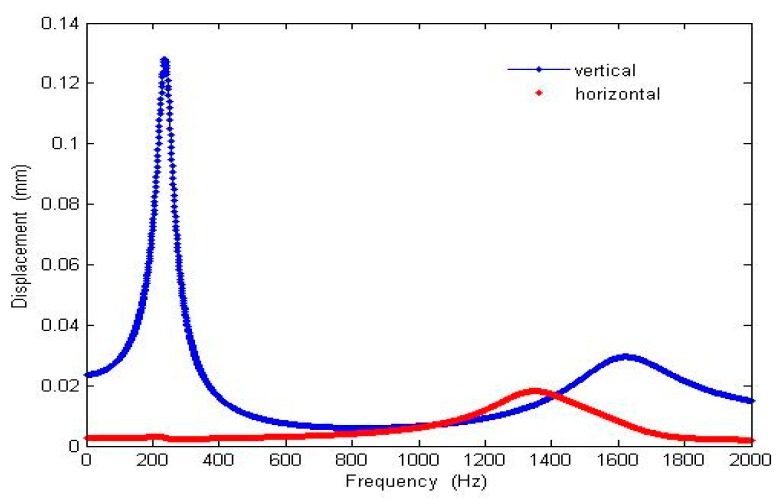
ANSYS Workbench harmonic responses of the optimized design with 60 µm × 60 µm × 4 µm proof mass, 1820 µm waveguide length and 20 µm slab width.

The result of the deflection angles of the optimized scanner is shown in Figure 8. The simulation shows that when the device is operating at 10 Hz in the non-resonant frequency direction (vertical direction) and 1.343 kHz in the resonant frequency direction (horizontal direction), the FOV are 16 and 15 mrad, respectively and a 130-line resolution at 0.08 Rayleigh damping and ±20 V input. 

The reduction in the proof mass from 300 µm × 300 µm × 24 µm in resonating design to 60 µm × 60 µm × 4 µm in non-resonating design was needed to simplify the fabrication and also to prevent the cantilever from bending too much due to the additional weight (Figure 19a). The width of the waveguide was also reduced from 55 to 20 µm to increase vibration in the horizontal direction.

In the parametric study, the geometry of the waveguide (the cross section and the length) dominated the line resolution of the scanner; the resolution increased with a flatter and shorter waveguide. The rib waveguide had a very similar resonant frequency response as long as the dimensions of the width of the ridge section were kept relatively small compared to the width of the slab section (<4×). All other geometric parameters did not appear to affect the line resolution significantly. In the analyses for the actuator and arm geometries, larger actuator pads allowed larger actuation; however, the sizes of the pads are limited by the design footprint (3 mm × 3 mm) defined for the previous endoscopic application. The largest angle of rotation achieved by the investigated push–pull designs was ~0.44 and 0.089 rad in vertical and horizontal direction respectively (for the resonating design) and 71 and 15 mrad, for the non-resonating design A shorter rotating arm appears to provide a larger angle of rotation. These findings suggest that the waveguide and the actuator layout can be considered separately to optimize the line resolution and scanning magnitude as we have shown in the analysis.

### 2.3. Optical Analysis

**Figure 9:**
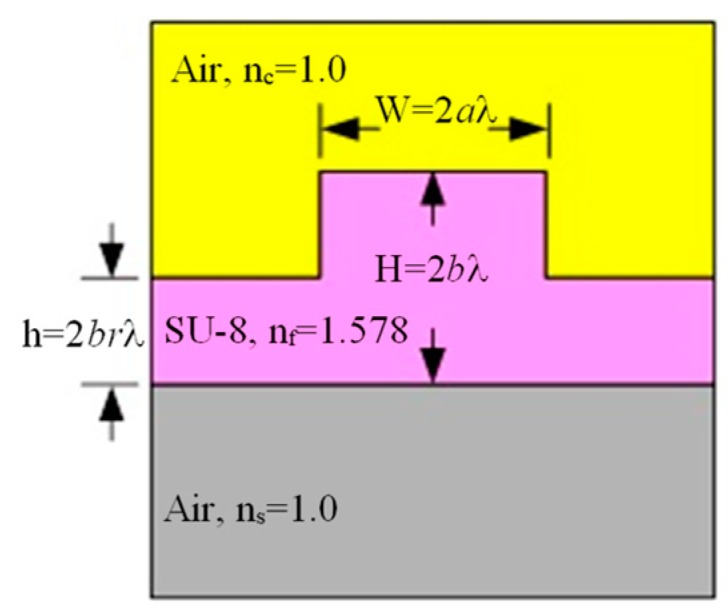
Cross section of the waveguide structure showing the geometric parameters and optical properties of the materials in each layer.

To optimize the light transmission and coupling between the tip of the input fiber and the cantilever beam requires modal analysis and investigation of the system’s coupling efficiency. The waveguide for the scanner has a rib waveguide feature (Figure 9), and it is made of an epoxy-based SU-8 negative-tone photoresist. The wavelength range required for the proposed display application is 400 to 650 nm. The final dimension of the waveguide structure was slightly different from the mechanical simulation. The slab was reduced to 20 microns and the proof mass also reduced to 60 μm × 60 μm × 4 μm to simplify the fabrication and improve dynamic performance of the cantilever vibration. One way to predict the dimensions of the rib waveguide structure was based on the single mode conditions proposed by Soref [26].

(4) $$\frac{H}{\lambda }\sqrt{n_{f}^{2}-n_{s}^{2}}\geq 1$$

(5) $$0.5\leq r\equiv \frac{h}{H}<1$$

(6) $$\frac{a}{b}=\frac{W}{H}\leq \left(\frac{q+4\pi b}{4\pi b}\right)\frac{1+0.3\sqrt{{\left(\frac{q+4\pi b}{q+4\pi rb}\right)}^{2}-1}}{\sqrt{{\left(\frac{q+4\pi b}{q+4\pi rb}\right)}^{2}-1}},$$

where *q* is defined as:

(7) $$q=\frac{\gamma_{c}}{\sqrt{n_{f}^{2}-n_{c}^{2}}}+\frac{\gamma_{s}}{\sqrt{n_{f}^{2}-n_{s}^{2}}},$$

where $\gamma_{c}=\gamma_{s}=1$ for transverse electric (TE) modes and $\gamma_{c}=\frac{n_{c}^{2}}{n_{f}^{2}}$ and $\gamma_{s}=\frac{n_{s}^{2}}{n_{f}^{2}}$ for TM modes.

It can be seen from equation 4 that $H\geq \frac{\lambda }{\sqrt{n_{f}^{2}-n_{s}^{2}}}$ for single mode operation. Therefore, H ≥ 0.314 µm at λ = 400 nm and H ≥ 0.531 µm at λ = 650 nm. 

**Figure 10:**
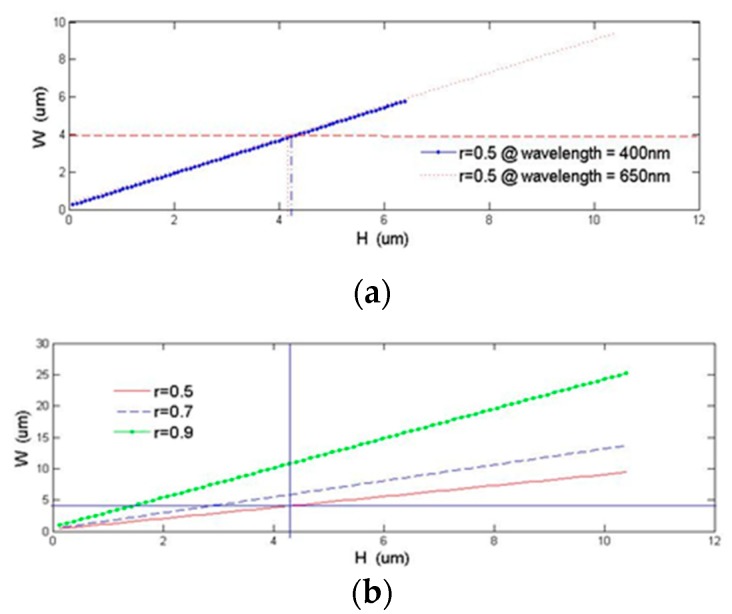
Waveguide width as a function of waveguide height at (**a**) λ = 400 and 650 nm and (**b**) at different r values at λ = 650 nm.

To maintain a single mode operation based on the above condition in TE polarization for any given *H*, the rib width *W* must be designed and operated less than or equal to the value on the curve (Figure 10). By fixing the ratio (*r*) between the overall height (*H*) and the slab height (*h*) to 0.5, we generate a list of waveguide dimensions that we can use for the scanner design (Figure 6 and Table 2. In case 1, the cross section of the rib waveguide has dimensions relative to the core diameter of a single mode fiber (2.9 to 3.9 µm mode field diameter for 400 to 600 nm wavelengths), which is an ideal geometry for the proposed end-butted coupling design. Assuming the same input applies at the center of the rib waveguide, the output power for different waveguide geometries is summarized in Table 2.

**Figure 11:**
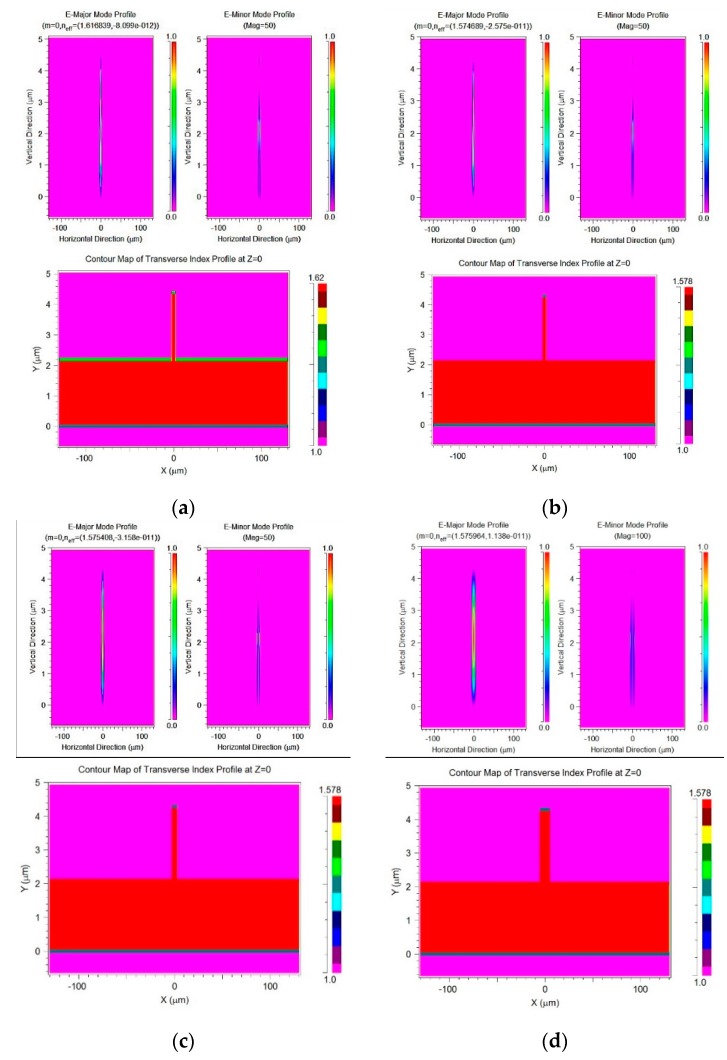
Index and modal profile of the proposed rib waveguide at λ = (**a**) 400 and (**b**) 650 nm with r = 0.5 and at 650 nm with (**c**) r = 0.7 (**d**) and r = 0.9 respectively.

Using modal analysis, based on a SU-8 (n = 1.578 at λ = 650 nm and n = 1.624 at λ = 400 nm [27]) rib waveguide structure with a rib width (*W*): waveguide height (*H*): slab height (*h*) ratio as shown in Table 2, the result confirms single mode operation for TE mode input with wavelengths between 400 to 650 nm. Figure 11 shows the index and modal profile of all three cases observed at 1.282 mm from the coupling end at λ = 650 nm (Figure 11b–d). The profile is similar at 400 nm (Figure 11a). Fields all appear confined and single mode. 

**Figure 12:**
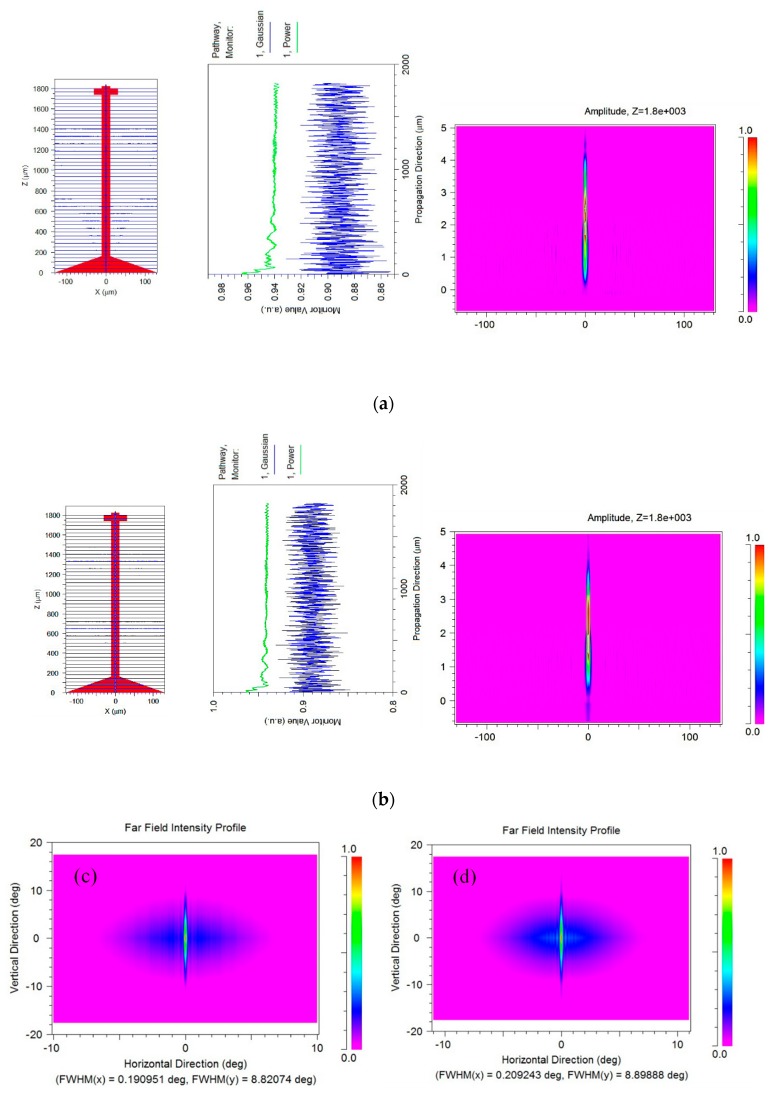
Wave propagation along the waveguide using a Gaussian beam profile for the 4 μm core input fiber (**a**) operating at 0.4 μm, (**b**) operating at 0.65 μm, middle figure showing field amplitude profile at z = 1800 µm (near the tip of the cantilever waveguide), (**c**) far field beam profile at λ = 400 nm and (**d**) 650 nm.

An optical simulation using Rsoft beam propagation program (BMP) software was performed to analyze the coupling efficiency. In order to simplify the analysis, scattering and absorption were neglected and it is assumed that the input to the waveguide is a single mode Gaussian beam. The numerical simulation is based on a SU-8 rib waveguide structure with a rib width: waveguide height: slab height ratio of 4:4:2. The results confirmed single mode operation for TE mode input with wavelengths between 400 and 650 nm. As shown in Figure 12, a very low light loss for wavelengths operates between 0.4 and 0.65 µm if center of the input mode field aligns with the center core of the waveguide (X = 0, Y = 2). For an input with a Gaussian profile, the coupling efficiency for both wavelengths was around 96.5% and 97% for λ = 400 nm and λ = 0.65 μm, respectively. Minimum light attenuation was observed along the taper and cantilever waveguide sections. The light throughput is roughly the same as the initial coupling efficiency (93.8% and 94.2% respectively). The same coupling efficiency and throughput was also observed if the input was a zeroth order mode. However, the alignment of the input with respect to the core of the rib waveguide greatly affects the coupling efficiency. Table 2 summarizes the output power as a function of input position operating at 650 nm wavelength. When fiber is vertical lateral displacement (y direction), the coupling loss was less significant than the horizontal displacement (x direction) as long as the beam was confined inside the waveguide. This is most likely the main contribution to the observed low coupling efficiency in the measurement. It is also shown in our previous multimode 100 µm × 85 µm × 2100 µm SU-8 rectangular waveguide that combine loss stem from mode coupling, scattering and absorption is around 28.6% [17,18]. The simulation also shows that the shape of the proof mass does not matter in the optical simulation, because as long as the input beam is confined inside the ridge area of the waveguide, single mode propagation is maintained. As shown in Figure 12a,b, both output fields at 400 and 650 nm appear to be single mode and nicely confined inside the waveguide. The far field intensity profile is also generated and match quite closely to the experiment result with ~ 0.22° in x direction and 8.8° in y direction FWHM at λ = 400 and 650 nm (Figure 8). As expected, the FWHM for 400 nm is slightly smaller than 650 nm. 

## 3. Fabrication

### 3.1. Scanner Fabrication Process

**Figure 13:**
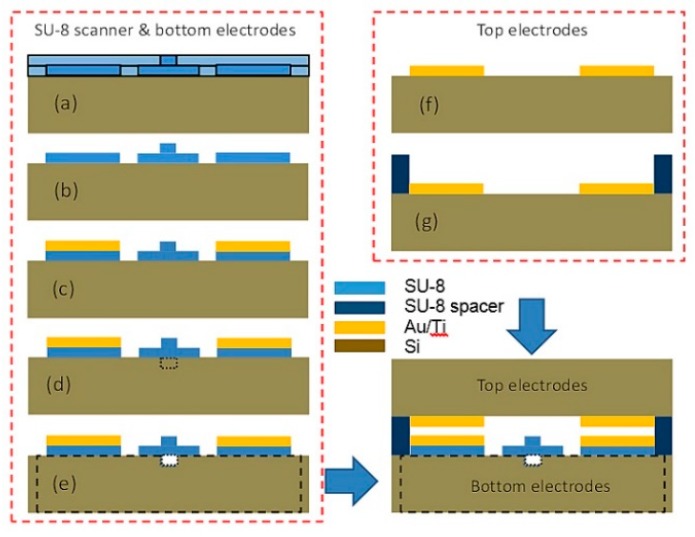
Fabrication process of electrostatic scanner. (**a**–**e**): scanner body and bottom electrodes (**f**,**g**): top electrodes. (**a**) Double-layer photolithography process to define rib shape waveguide and scanner pads; (**b**) Development process to create SU-8 waveguide and body; (**c**) Au/Ti thin films are deposited and patterned by lift-off process; (**d**) Front side fiber grooves are patterned and deep etched using deep reactive-ion etching (DRIE); (**e**) the scanner (bottom electrode and waveguide) were released by DRIE again from the backside of the wafer; (**e**) Backside etch-through to release actuators and waveguide; (**f**) top electrode is deposited and patterned on a second silicon wafer; (**g**) SU-8 spacers are spun and patterned.

The fabrication procedure of the electrostatic scanner is shown in Figure 13 [22]. A 4-inch silicon wafer with a standard thickness of 525 ± 25 μm served as the substrate for the device fabrication. The substrate was immersed in a Piranha solution (H2SO4:H2O2 = 4:1) to clean off organic residues and it was rinsed thoroughly using deionized (DI) water. After rinsing, the substrate was dried using spin rinse dryer (SRD) and then it is further dried on a hotplate at 200 °C for 10 min. After cleaning, a double layer SU-8 structure was created on the substrate using photo-lithography process. A 2 μm layer of SU-8 5 (MicroChem, MA) was first spun coat onto the silicon substrate to form the actuator body and slab part of the waveguide following instructions provided by the MicroChem Corp. An additional layer of 2 μm-thick SU-8 layer was spun and exposed on top of the first layer after post-exposure bake (PEB) to create the rib of the waveguide. During pre-baking, the temperature was first held at 70 °C for one minute before ramping from 70 °C to 105 °C at a rate of 3 °C/min to reduce the extrinsic stress of the SU-8. Finally, the sample was held at 105 °C for 15 min before cooling down to room temperature at a rate of 2 °C/min. 

Using a contact aligner, the geometry of the waveguide slab, the rib, and the actuator body were transferred from the soda lime masks onto the double-layer SU-8 film via UV exposure. The exposed film underwent the post exposure bake (PEB) to cross-linked the defined geometry. During PEB, the wafer was first placed on a hotplate for 1 min. at 70 °C. The temperature was held for 1 min, and then it was ramped from 70 °C to 105 °C at a rate of 3 °C/min. The hotplate was held at 105 °C for 1 min, and then it was cooled to room temperature over a 30-min period (a longer cooling period compared to manufacture specification). After the sample was cooled down to room temperature, the double-layer SU-8 film was then developed in PMGEA (an ethyl lactate and diacetone alcohol, MicroChem Corp., Westborough, MA, USA) for 1 min with mild agitation. The wafer was rinsed thoroughly in isopropyl alcohol (IPA) for another 1 min and then it was dried using SRD. The 20 nm Ti with 200 nm Au metal electrodes were made using E-beam deposition and lift-off process. Prior to metal deposition, a layer of negative photoresist NR9-3000PY (Futurrex Inc, Franklin, NJ, USA) was spun onto the SU-8 layer. The pattern was then transferred from a soda lime mask to the resist to define the electrode area. Similar to the previous process, the fiber groove was formed through subsequent photolithography and DRIE. Finally, the device (scanner and bottom electrodes) were released from backside via bulk silicon etching using DRIE.

The 20 nm Ti with 200 nm Au metal electrodes were made using E-beam deposition and lift-off process. A layer of negative photoresist NR9-3000PY (Futurrex Inc) was spun onto the SU-8 layer before the metal deposition. The pattern was then transferred from a soda lime mask to the photoresist to define the electrode area. Similar to the previous process, the fiber groove was formed through subsequent photolithography and DRIE. Finally, the device (scanner and bottom electrodes) was released from backside via bulk silicon etching using DRIE. 

The fabrication process of the top electrode was a two-step micro-fabrication process. A second four-inch wafer was prepared as a substrate for SU-8 deposition as before. The top electrode area is defined using photolithography process, and an e-beam evaporator system is used to deposit the Titanium adhesion layer and the Au electrode. The metal thin films were then patterned by lift-off process. A SU-8 spacer was spun and patterned onto the electrode layer. With the aid of an extra packaging design, which will be introduced in the next section, the top electrodes were aligned with the scanner and the bottom electrodes. 

### 3.2. Device Package

**Figure 14:**
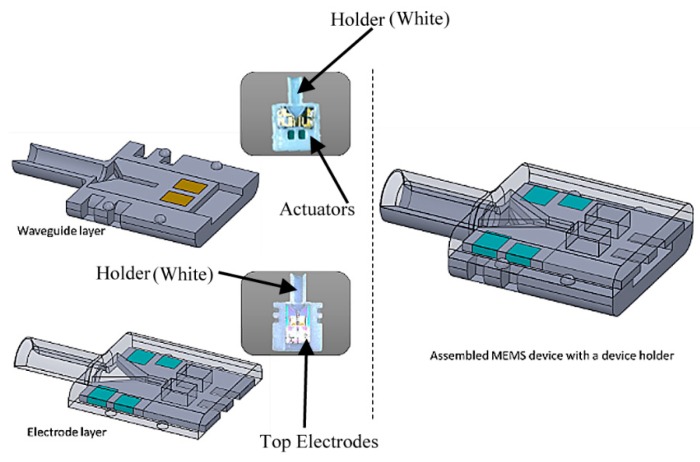
Schematics of assembled MEMS device, including top electrode, scanner, bottom electrodes, and gradient index (GRIN) lens with the device holder.

An 8.0 mm × 8.0 mm × 1.0 mm acrylic-based photopolymer holder was designed and fabricated using a multi-material three-dimensional printing system (X-axis: 600 dpi; Y-axis: 600 dpi; Z-axis: 1600 dpi, Connex350, Stratasys Ltd.) [22] to secure the MEMS device. A gradient index (GRIN) lens (4.85 mm length × 2 mm diameter GRIN lens rod by GRINTECH GmbH, Jena, Germany, working distance = 20 mm, beam width = 20 µm, view angle = ±30°, and NA = 0.5) was installed and secured in a tube structure formed by the assembly of the upper and the lower part of the holder as shown in Figure 14. Our proposed GRIN lens was fixed and placed at a predetermine distance beyond the end of the cantilever inside the holder to focus the diverging beams at the output of the waveguide to an object plane at a certain distance away. The estimated beam width at the focal plane was around 20 µm when the GRIN lens was placed at 0.4 mm away from the waveguide at the scanner designed wavelength of operation. The calculated angular deflection of the proposed scanning system was about 5° total (2.5° on each side), which minimizes aberrations and vignette at the output. 

## 4. Results and Discussions

**Figure 15:**
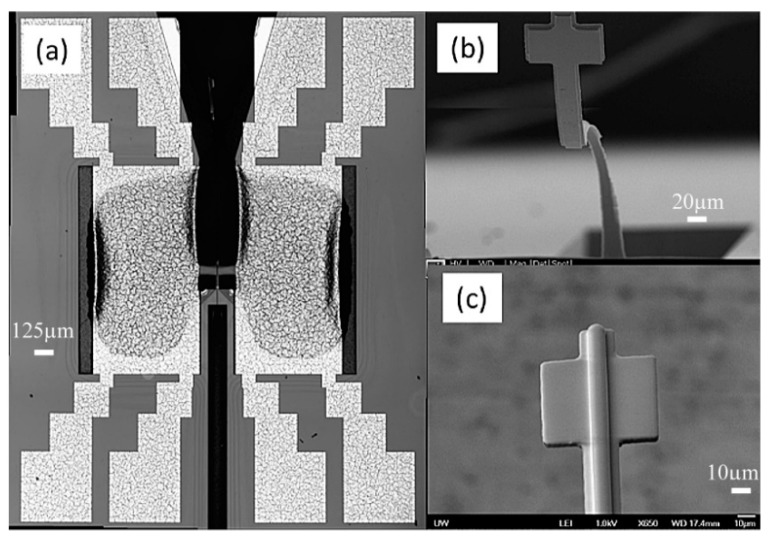
(**a**) MEMS scanner with push-pull actuators. (**b**) Bending waveguide due to large aspect ratio and residual stress. (**c**) SU-8 rib waveguide at the tip.

**Figure 16:**
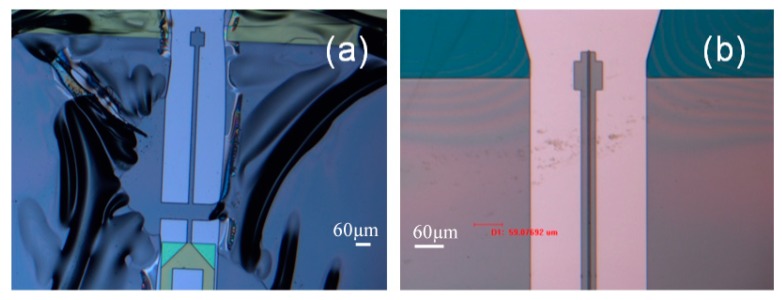
Lithographic result of double-layer SU-8 waveguide on nitride/silicon substrate. (**a**) Baking at lower temperature (65 °C /95 °C) where large SU8 film area such as actuators appear to peel from the nitride surface (**b**) when operates at higher temperature (70 °C/105 °C), SU8 film appears smooth and laminated.

The device was successfully fabricated (Figure 15a,c) [22]. Initially, due to its large aspect ratio and residual stress, the cantilever waveguide appeared bent (Figure 15b) [22]. This problem was quickly resolved by carefully controlling the baking procedure. Using more gradual heating and cooling and different baking temperatures, we were able to obtain a straighter beam (Figure 19a) [22]. To resolve the adhesion problem (shown in Figure 16a) [22] between the SU-8 waveguide and the silicon nitride substrate, the soft bake temperature was increase from 65 °C to 70 °C (soft bake) and the post exposure bake (PEB) temperature was increased from 95 °C to 105 °C. The increase in temperature produced a smooth and laminated surface on the SU-8 layer (Figure 16b) [22]. The dimension of the waveguide was slightly reduced in thickness and width due to the double layer structure usually being produced much thinner. 

**Figure 17:**
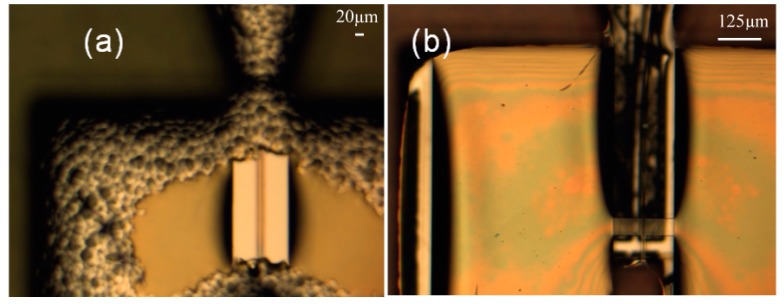
(**a**) The 750-deep reactive ion etching cycles were used to release the scanner (picture is taken from the backside), (**b**) 30 additional cycles were later added to further remove the Si residue around the edges of the window.

The devices were released using backside deep reactive-ion etching (DRIE). However, it was harder to etch around the corner, leaving silicon residue (Figure 17a) [22]. When more etching cycles were added, the corners became clear, but some of the SU-8 waveguide did not survive (Figure 17b) [22]. In the future, we will revise the mask design so that SU-8 waveguides are better protected during the DRIE. 

**Figure 18:**
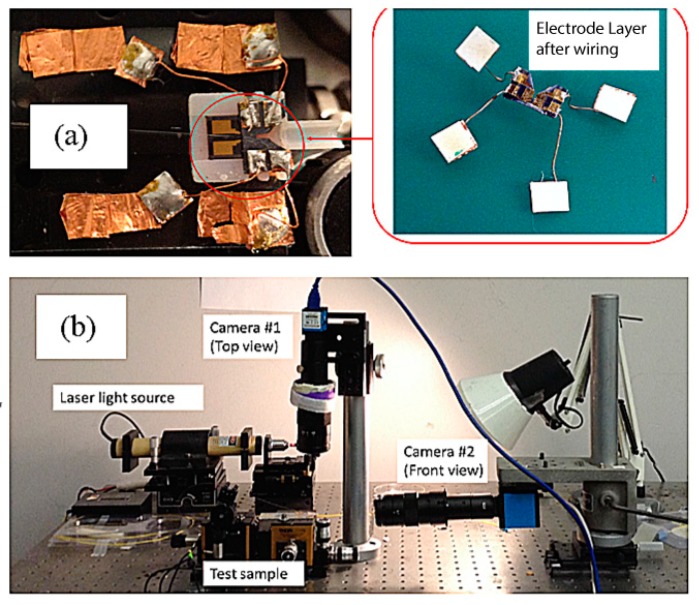
(**a**) MEMS device test sample: device in the red square shows the top electrode. The bottom electrode consists of the moving push-pull actuators and the cantilever waveguide (partially covered by the top electrode), (**b**) optical tests were taken by two cameras equipped with high power lenses (250× magnification).

The scanner test sample setup is shown in Figure 18 [22]. The top and bottom layers of the scanner were secured inside the rapid-prototyped holder and larger external contact pads were built to allow easier access to the smaller device contacts (Figure 18a). A 10 mW He–Ne laser (λ = 632.8 nm) provides the input light to a single mode optical fiber (diameter = 4.3 µm). A XYZ positioning stage (incremental linear encoder with 100 nm resolution) provides the accurate light coupling between the light-carrying optical fiber to the SU-8 rib waveguide. Two cameras equipped with high power lenses (250× magnification) were used to aid the alignment of the optical fiber in the vertical and horizontal directions. A third camera, a microscope, is placed near the tip of the waveguide to observe the light emitted.

**Figure 19:**
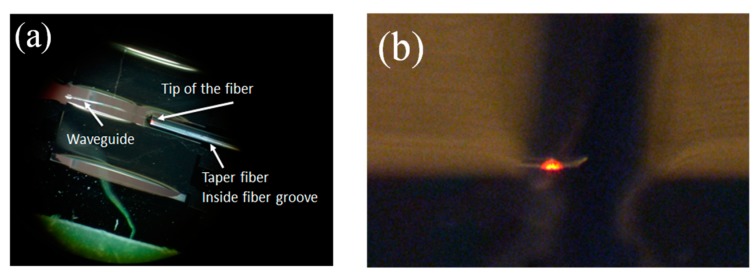
(**a**) Optical coupling from the taper fiber to the SU8 rib waveguide. (**b**) Light observed at the tip of the SU8 rib waveguide. Light appears nicely confined inside the core of the waveguide.

After using the microscope and the cameras to align the fiber to the scanner, light can be observed at the tip of the waveguide (Figure 19), Figure 19a shows the aerial view of the light coupling from taper fiber to the rib waveguide. Light can be seen at the tip of the waveguide. Some coupling loss can be seen from the escaping light at the interface. Some dimmer escaping light can also be observed at the bending part of the waveguide. Despite the losses, Figure 19b shows that the optical beam is nicely confined by the core of the waveguide. The intensity of the light at the output of the waveguide and the light from the input fiber were measured. The average coupling (or transmission) efficiency was measured to be around 10% (Table 3). The low coupling efficiency was mainly due to inherent process imperfections in making the fiber groove. 

**Figure 20:**
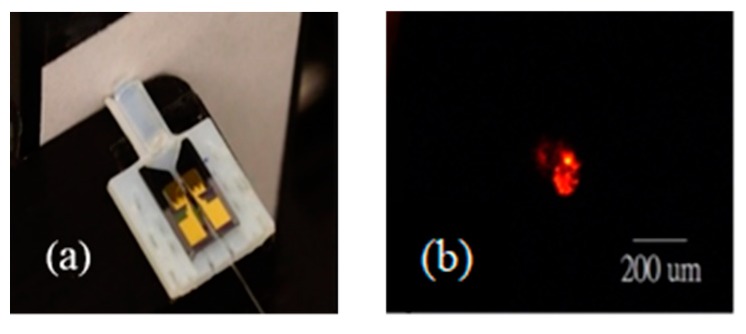
Pictures show (**a**) scanner coupled with optical fiber and GRIN lens. (**b**) direct observation of beam profile. Beam spot is the bright spot in the figure. Surround scattering red lights are from input fiber.

The optical test was also performed on scanner to measure the beam profile coming from the GRIN lens coupled device package (Figure 20). A 20-micron beam diameter is observed at 2 cm focal length, matching the estimated beam width.

**Figure 21:**
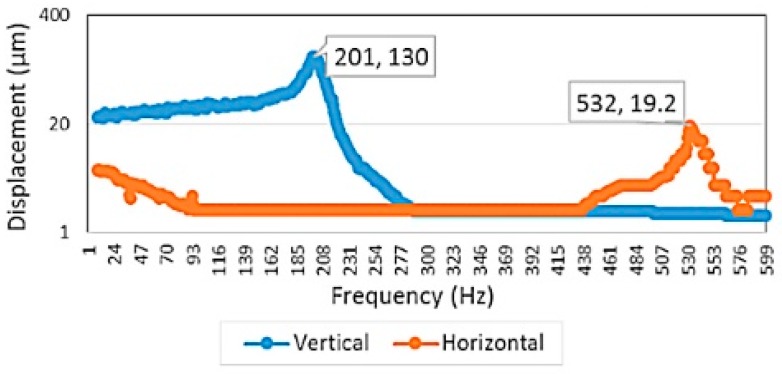
Waveguide mechanical frequency response. Both vertical and horizontal displacement were measured. Driving input voltages are 100 V DC applied at the bottom electrodes and +150 V AC at the top electrodes.

The performance of the device was evaluated by applying voltage according the patterns shown in Figure 2. Vertical scanning motion of the waveguide was accomplished by applying 100 VDC to the bottom electrodes and ±150 V AC voltage to all of the top electrode with synchronized phase. Horizontal scanning motion of the waveguide was achieved by driving actuators with the same voltage settings for generating vertical motion but with a 180° phase delay between the left and right top electrodes. The driving voltages were much larger than the estimated voltages due to the increase in gap space between top and bottom electrodes compared to the original design. To find the resonant frequencies for both directions of the waveguide motion, the operating frequency was swept from 1 to 10,000 Hz (1 to 6000 Hz is shown here) (Figure 21) and the displacement of the waveguide was measured. A vertical resonant mode was found at 201 Hz, which was slightly lower than the simulation result (240.07 Hz). A horizontal mode was found at 535 Hz, compared to 1343.82 Hz in the simulation. The amplitudes of vibration were 130 and 19.2 µm, respectively. The initial displacement measurement and actuation displacement of the scanner was smaller than expected, most likely due to inaccurate modelling of air damping and boundary condition, intrinsic stress in the cantilever and the under etched residual silicon around edges of the actuator (Figure 26). 

**Figure 22:**
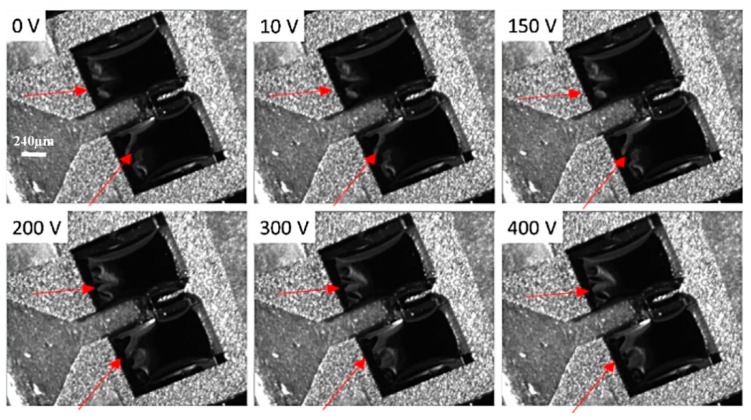
Actuator deformation under different DC applied voltages. Deformation can be observed by comparing the change in areas indicated by the red arrows.

Due to larger air gap (compared to the simulation), additional DC voltage was added to reduce the input driving voltage. Figure 22 [22] shows the behavior of actuator membrane when various DC voltages were applied to the top electrode while bottom electrodes were grounded. The deformation of the actuator membrane can be observed visibly starting at 200 V. 

**Figure 23:**
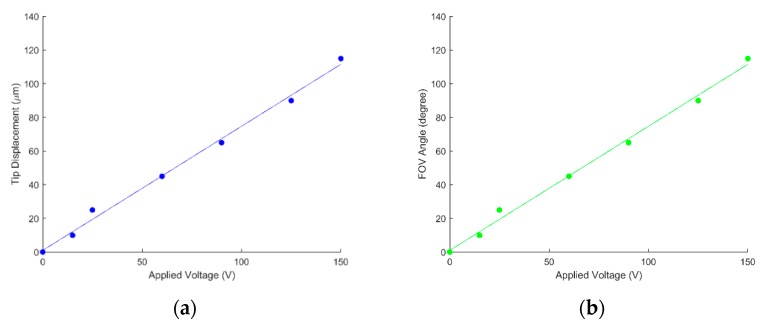
(**a**) Waveguide tip displacement and (**b**) corresponding scanning angle vs. applied voltage at 1 Hz driving frequency.

**Figure 24:**
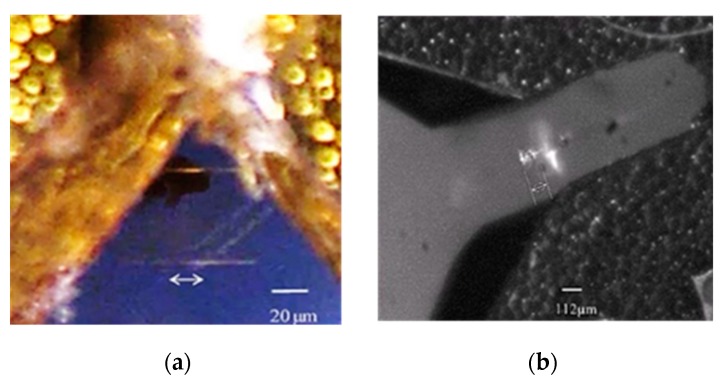
Pictures show superimposition of waveguide’s peak displacement in (**a**) horizontal and (**b**) vertical direction. Both displacements are operated at 1 Hz ± 150 V AC and 100 V DC. Black spot on proof mass in left image is the silicon residue.

To characterize the scanning performance, the tip displacement and the scanning angle of the waveguide were measured against input actuation voltages (Figure 23) [22]. The scanning angle was calculated as the inverse of the sine function of the tip displacement over the waveguide length, in this case, 1820 µm. An amplifier (30×) was used in order to create a large enough electric field for driving the device. A linear relation can be observed between the tip deflection/scanning angle and the driving voltage. A tip displacement of 111.8 µm (vertical) and 20 µm (horizontal) was observed at the maximum test voltage of 150V under 1 Hz driving frequency (Figure 24). 

**Figure 25:**
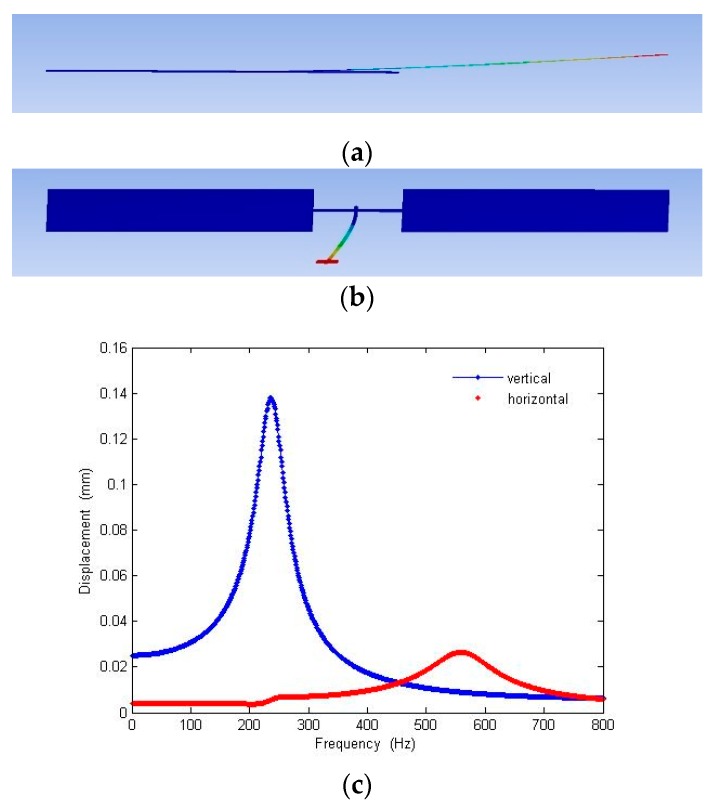
ANSYS modal analysis of scanner with slab width reduce to 10 microns. The two lowest frequencies 238.32 Hz and 562.68 Hz in the simulation appear closely matching the experiment results. (**a**) Mode shape at 238.32 Hz, (**b**) mode shape at 562.68 Hz. (**c**) Corresponding harmonic responses measured experimentally.

The frequencies of the two lowest vertical and horizontal modes are 240.07 and 1343.8 Hz in non-resonating simulation, compared to 201 and 532 Hz in experimentation. (Figure 21). The discrepancies were most likely due to reduction in the overall geometry in the final device. Due to heat related shrinkage, with 10% deviation in the width and/or thickness, overexposure, and process errors, the resonant frequency can vary by more than 10%. According to the results obtained from our later ANSYS simulation, where by decreasing the slab width from 20 to 10 microns, the two lowest resonant frequencies then matched closely to the experiment results. In this case, the vertical and horizontal frequencies in the simulation were then 238.32 and 562.68 Hz, respectively (Figure 25).

**Figure 26:**
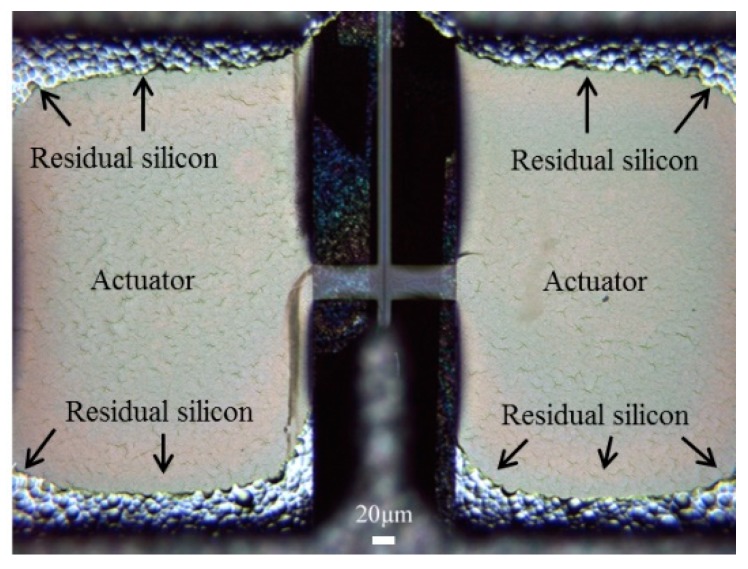
Arrows in the microscopic picture show the residual silicon around corners of the actuators after DRIE. Picture taken from the backside of the actuators.

The other factors could be stem from imperfections in the fabrication process. For example, varying thickness in the (optimally uniform thickness) SU-8 waveguides, additional under-etched silicon residues on the SU-8 after the DRIE backside etching (see the dark spot (silicon residue)) on the backside of the proof mass in Figure 24 and vibration reduction in actuators due to the silicon residue on the edges of the backside window in Figure 26. 

**Figure 27:**
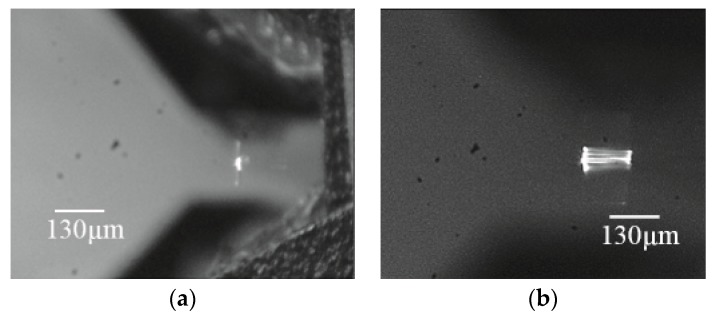
Microscopic images show the vertical displacement driving at (**a**) 0 Hz, (**b**) non-resonating 2D scanning at 201 Hz in vertical and 20 Hz in horizontal direction. (Pictures are taken directly above the scanner so vertical and horizontal directions are switched).

A 2D scanning motion is successfully demonstrated in the non-resonating configuration with 201 Hz in vertical direction and 20 Hz in horizontal direction (Figure 27). The 34 µm horizontal and 130 µm vertical displacement or 0.019 to 0.072 radians in field of view were obtained. Here the non-resonating mode was performed in the horizontal direction instead of vertical direction as described in the earlier non-resonating simulation because the experiment shows a much improve overall dynamic performance (a larger vertical and horizontal mechanical vibration or FOV) (Figure 21) [22].

## 5. Conclusions

In this paper, we presented a novel, fully integrated MEMS-based 2D mechanical scanning system using 1D push–pull actuators. Detail design, fabrication and tests were performed. The results from the preliminary tests prove the push-pull actuator concept works and two-directional displacements is possible using this new 1D actuator design. 

The scanner’s mechanical and optical parameters were analyzed and optimized to determine the best line resolution and FOV. Based on the simulation, the dominant factors affecting scanner resolution are the cross-section and length of the waveguide. Higher resolutions can be achieved with a vertically thin or horizontally wide T cross section on a longer waveguide. The best optical single mode operation can be achieved by rib waveguide configuration.

The horizontal resonance of 532 Hz and vertical resonance of 201 Hz were found from the mechanical test. Discrepancy to the simulation results as mentioned in the discussion are mainly due to the dimension reduction in the proof mass and waveguide slab width and thickness. As shown in Figure 25, reduction in proof mass (5×) increases the vertical vibration resonant frequencies (4×) and smaller slab width (2.2×) decreases the horizontal resonant frequencies (2×). It is worth noting that it is extremely difficult to model this scanner design due to the fact that both optical and mechanical structure must both be optimized at the same time. It is also a challenge to correctly model the residual stress and damping and other factors that can only be found experimentally. Other factor contribute to the deviation could be result of fabrication errors. With 10% deviation in the width and/or thickness, the resonant frequencies can vary by more than 10%. The under-etched silicon remained in the backside of the SU-8 waveguide can add additional mass or stiffness to the system and further vary the resonant frequencies. 

Initial coupling tests showed a 10% coupling efficiency between the optical fiber and the MEMS waveguide. The measured value is relatively low compared to the optical simulation results. The relatively large cross section (4 μm in height and 20 μm in width) of the double-layer SU-8 rib waveguide design is expected to provide a coupling efficiency of ~94% with a Gaussian beam profile input and broad band single mode operation (λ = 0.4 to 0.65 μm) with a minimal transmission loss (3% output transmission loss–model not including absorption and scattering loss). The coupling efficiency decrease is likely to be caused by imperfections in the fabrication process used during the process and the micron-range precision needed to align the optical fiber with the waveguide coupler for optimal coupling. As shown in simulation, slight offset in input position in either the vertical or horizontal direction creates tremendous coupling loss (e.g., a 1 micron offset from the original x = 0 μm and y = 2 μm input position results in an observed 17% light reduction). 

For the FOV and line resolution test, the largest FOV is found to be 0.015 rad at 532 Hz in horizontal and 0.072 rad at 201 Hz in vertical direction (Figure 21) and the largest line resolution is calculated to be about 90 dots per line. The scanner system can definitely be improved. More revisions to the actuator and coupling system are needed to achieve a better FOV and line resolution. 

A 2D scanning motion is successfully demonstrated in the non-resonating configuration with 201 Hz in vertical direction and 20 Hz in horizontal direction. A 34 µm horizontal and 130 µm vertical displacement or 0.019 to 0.072 radians in field of view were obtained.

In this paper, we have successfully demonstrated a 2D optical scanner using pairs of 1D electrostatic parallel plate push-pull actuators. Results show tip displacement changing linearly as a function of input actuator voltage. The light beams are well guided and confined within the core of the rib waveguide structure. The optical package with GRIN lens also focuses the output diverging beam as intended. A 2D scanning motion operating at non-resonating configuration is also successfully demonstrated.

## Figures and Tables

**Table 1:** Resonant frequencies obtained by analytical estimation, finite element methods (FEM), and Architect3D.

		Analytical	FEM	Architect3D
X-axis	1^st^	58.5	58.3	55.9
	2^nd^	743	630	707
Y-axis	1^st^	1113	1103	1062

Unit for frequency measurements is in Hz.

**Table 2:** Comparison of output power as a function of input beam position and waveguide geometry.

	r	W	H	h	Center of Input	Output Power
Case 1	0.5	4.045	4.290	2.145	X = 0, Y= 2.0	94.2%
Case 2	0.5	6.097	6.630	3.315	X = 0, Y = 3.0	90%
Case 3	0.5	8.036	8.840	4.420	X = 0, Y = 4.0	77%
Case 4	0.7	5.851	4.290	2.145	X = 0, Y = 2	95%
Case 5	0.9	10.755	4.290	2.145	X = 0, Y = 2	96%
Case 6	0.5	4.045	4.290	2.145	X = 0, Y = 1	77%
Case 7	0.5	4.045	4.290	2.145	X = 0, Y = 3	76%
Case 8	0.5	4.045	4.290	2.145	X = 0, Y = 4	46%
Case 9	0.5	4.045	4.290	2.145	X = 1, Y = 3	60%
Case 10	0.5	4.045	4.290	2.145	X = 2, Y = 3	33%
Case 11	0.5	4.045	4.290	2.145	X = 20, Y = 1	0.5%
Case 12	0.5	4.026	4.40	2.20	X = 0, Y = 2.0	93.8%

Case 1 to 11 are waveguide geometry and corresponding output power at 650 nm. Case 12 is operating at 400 nm (unit: μm).

**Table 3:** Light coupling measurements.

	Intensity Measured at Input Fiber End (V)	Intensity Measured at Waveguide Output (V)
mean 1	0.077	0.014
mean 2	0.079	0.004
mean 3	0.075	0.006
Mean	0.077 ± 0.001	0.008 ± 0.001
Coupling efficiency (%)	N/A	10.390
